# The perceived effects of COVID‐19 while living with a chronic illness

**DOI:** 10.1111/jnu.12835

**Published:** 2022-10-25

**Authors:** Mary Ryder, Suzanne Guerin, Rita Forde, Grainne Lowe, Tiny Jaarsma, Madeline O'Neill, Carmel Halley, Michael Connolly

**Affiliations:** ^1^ School of Nursing, Midwifery & Health Systems University College Dublin Dublin Ireland; ^2^ School of Psychology University College Dublin Dublin Ireland; ^3^ Nursing, Midwifery and Palliative Care Kings College London London UK; ^4^ Institute of Health and Wellbeing Federation University Australia Melbourne Australia; ^5^ Department of Health, Medicine and Caring Sciences Linköping University Linköping Sweden; ^6^ Julius Center University Medical Centre Utrecht Utrecht The Netherlands; ^7^ School of Nursing, Midwifery & Health Systems, University College Dublin Registered Advanced Nurse Practitioner Our Lady's Hospice and Care Services, Harold's Cross Dublin 8 Ireland; ^8^ St Vincent's University Hospital Dublin 4 Ireland; ^9^ School of Nursing, Midwifery & Health Systems University College Dublin and Our Lady's Hospice & Care Services Dublin Ireland

**Keywords:** arthritis, chronic illness, COVID‐19, diabetes, heart failure, lung disease, self‐care

## Abstract

**Introduction:**

A diagnosis of chronic illness posed a serious threat to people during the recent COVID‐19 pandemic. People with chronic illnesses were faced with increased mortality and reduced access to healthcare. Self‐care is the process of maintaining health and managing a chronic illness. Nurses working in specialist services provide healthcare education to people with chronic illnesses. Access to these nurses was decreased during periods of the COVID‐19 virus escalation due to the reconfiguration of services and redeployment of nurses. The purpose of the research was to learn from the experiences of people with a chronic illnesses in self‐care behaviors and accessing altered healthcare services to inform future practices.

**Design:**

A population survey design.

**Methods:**

A mixed methods survey was designed, combining validated questionnaires and scales with open‐ended questions. A convenience sample was utilized via using social media platforms. Data analysis included descriptive and inferential statistics. Content analysis was used to analyze open‐ended responses.

**Results:**

There were 147 responses, with approximately half reporting no changes in face‐to‐face healthcare contact, 41% reporting decreased contacts and 12% increased contacts. Non‐face‐to‐face contacts were reduced by almost 9%, did not change by almost 60%, while 33% indicated an increase. Participants reported mixed perceptions in contact with healthcare providers during restrictions. In the Patient Assessment of Chronic Illness Care and the Self‐Care of Chronic Illness scales, participants scored statistically lower scores than in previous studies. Participants indicated that public health restrictions negatively impacted their confidence, created challenges with re‐engaging and that access to care was more difficult.

**Conclusion:**

This research highlights the importance of providing continued support to people with chronic illness irrespective of other challenges to healthcare services. A structured approach to virtual self‐care education is required.

**Clinical relevance:**

This research concluded that the experience of access to one healthcare professional as opposed to diverse multidisciplinary input was similar for a number of chronic illnesses groups of people during the COVID‐19 pandemic. There was an altered dynamic of virtual contacts with healthcare providers and a lack of confidence interpreting what monitoring was required by people with a chronic illnesses due to a lack of preparedness for virtual healthcare delivery.

## INTRODUCTION

Coronavirus disease is an infectious disease commonly associated with infections in the nose and throat. In early 2020, the World Health Organization (WHO) identified SARS‐CoV‐2 as a new type of coronavirus disease (COVID‐19) which quickly spread throughout the world, through traditional person‐to‐person contact (WHO 2020) causing viral pneumonia.

In people with a diagnosis of diabetes, the risk of death associated with COVID‐19 infection was significantly increased (Chee et al., 2020; Hillson, 2020). While international trends demonstrated a decrease in hospitalizations for acute decompensated heart failure (Cox et al., 2020), people with heart failure were associated with increased mortality following a COVID‐19 diagnosis (Rey et al., 2020; Tomasoni et al., 2020). In addition, medications used to treat people with rheumatoid arthritis and lupus placed people at an increased risk of infection (Michaud et al., 2020). A diagnosis of a chronic illness posed a significant morbid threat for people causing distress (Antony et al., 2020). Yet access to chronic illness services was severely disrupted during restrictions.

The treatment and management of people with chronic illness traditionally include regular hospital visits with specialist healthcare teams, including medication titration regimens, and rapid consultation and review for reported symptoms of deterioration. Self‐care is a foundation for chronic illness management. Self‐care is defined as ‘a process of maintaining health through health promoting practices and managing illness’ (Riegel et al., 2012 p195) self‐care is performed in both healthy and ill states. Self‐care education empowers people with chronic illnesses to maintain stability and to assess and respond to indicators that their illness has become unstable (De Maria et al., 2021). The primary role of nurses working in specialist chronic illness services is to deliver self‐care education to people. Self‐care has traditionally been delivered by nurses in a face‐to‐face format and is supplemented with written and digital materials.

Teaching self‐care is a complex phased process and evidence indicates self‐care improves over the first year of the illness (Aamodt et al., 2020). Multidisciplinary teams in chronic illness management are associated with improved outcomes for people attending, including increased compliance, reduced anxiety, and a reduction in hospitalizations. During the acute phases of the pandemic, people with chronic illness faced an increased risk to their health due to abrupt changes in healthcare delivery, to virtual contact service.

A significant proportion of the literature to date reports healthcare professionals' perceptions of the effect of COVID–19 on specialist patient populations (Ciacci & Siniscalchi, 2020; Forde et al., 2021; Mauro et al., 2020; Rey et al., 2020; Zukowski et al., 2021). There is a dearth of literature reporting people's perceptions of the effect of COVID‐19 on self‐care for their chronic illness. This research aims to explore the perceived effects of COVID‐19 on those living with a chronic illness during the pandemic and any associated restrictions. This research will provide insight into challenges faced by individuals when accessing the altered healthcare service delivery during the pandemic. The study will also seek to explore if new health‐seeking behaviors emerged during the COVID‐19 pandemic. The purpose of the research is to learn from the experiences of people with a chronic illnesses in self‐care behaviors and accessing altered healthcare services to inform future practices. The research questions included:

1. How did contact with healthcare change for people with a chronic illness during the COVID‐19 pandemic?
2. What was the self‐care experience for people with a chronic illness during the COVID‐19 restrictions?
3. How has COVID‐19 impacted the lives of people with a chronic illnesses?

## DESIGN

### Materials and methods

The target population included individuals living with a chronic illness, specifically diabetes, heart failure/cardiac conditions, arthritis, and respiratory conditions. The study used a population survey design, with all members of the target population invited to take part in the study. Participants that did not identify a chronic condition in their response were excluded from the study. To facilitate participation, an online/cloud‐based survey was created. The survey used mixed methods, combining validated questionnaires and scales and open‐ended qualitative questions. Participants were required to read the information page and select the option ‘I consent to participate’ to proceed to the survey.

A convenience sample was generated using social media recruitment methods. The study was advertised on Twitter, Facebook, and LinkedIn social media sites during May 2021. Relevant patient advocacy groups were tagged in the posts to raise awareness of the study.

### Data collection

Data were collected via a Qualtrics online survey. The completed questionnaire data were anonymous, and any identifiable data returned as part of open‐ended questions was de‐identified prior to analysis. Participants were asked to name all of the chronic illnesses that they had been diagnosed with. Questions exploring pre‐pandemic healthcare were phrased “in the year before the pandemic.” Questions exploring participants' responses during periods of restrictions were phrases “during the last six months” reflecting the period of restrictions before May 2021. Quantitative content included structured questions on the frequency of contact with professionals and three validated tools:

1. Self‐care of chronic illness inventory version 3, English version (Riegel et al., 2018). This is a self‐report measure of the processes of self‐care, designed for use with individuals with a range of chronic conditions. The scale has shown good reliability and validity in empirical studies (e.g., Brito Villa et al., 2022). The version used requested reports of both common and recently used monitoring behaviors and allows for the calculation of a standardized score out of 100 (higher scores indicate more frequent use of the behaviors) across four subscales assessing aspects of self‐care, monitoring, and control. Cronbach alpha values in the present study ranged from 0.428 to 0.871.
2. Patient assessment of chronic illness care tool (PACIC; Glasgow, Wagner, et al., 2005). This self‐report measure was designed as a brief assessment of patient‐reported care in the context of the Chronic Care Model. The five subscales and overall score allow for the calculation of an average score between 1 and 5, with higher scores indicating more positive reports. The PACIC tool has been widely used and shown to have good reliability and validity (e.g., Schmittdiel et al., 2008). Cronbach alpha values for the subscales in the present study ranged from 0.768 to 0.846, while the overall score had an alpha of 0.949.
3. Self‐care self‐efficacy scale (Yu et al., 2021). Confidence in self‐care was assessed using the 10‐item Self‐Care Self‐Efficacy scale, which was part of the Self‐Care of Heart Failure Index (Riegel et al., 2009). This brief scale has been used as a separate scale with individuals with a range of chronic illnesses, with scores ranging from 10 to 50, with higher scores indicating greater self‐reported self‐efficacy or confidence in managing self‐care. There is evidence of good reliability and validity (e.g., Osokpo et al., 2022) and Cronbach's alpha in the present study was 0.922.
4. Coronavirus impact scale (Stoddard et al., 2021). Given the relatively recent emergence of COVID‐19, this scale was chosen as a brief self‐report measure of the impact of COVID‐19, with the initial evidence of its reliability and use with a range of clinical samples (Stoddard et al., 2021). It generates a single score ranging from 8 to 32, with higher scores indicating a higher perceived impact associated with COVID‐19 and its associated restrictions. The authors note it is included in the NIH OBSSR suite of common instruments. Cronbach's alpha in the present study was 0.704.

The qualitative component used open‐ended questions to explore perceptions of changes in contact with healthcare providers during the pandemic and the impact on their life. An example of open‐ended questions include “Please describe what you feel have been the main changes to your contact with your specialist healthcare provider?” and “Please tell us about any other ways the coronavirus pandemic has impacted your life.”

### Data analysis

Data analysis included frequency analysis and descriptive statistics for core quantitative elements of the survey. In addition, inferential statistics included one‐sample t‐tests comparing scale scores to published means to allow the study to profile the sample against other chronic illness samples, and Pearson's correlations exploring relationships between scale scores. The impact of demographic variables was assessed using t‐tests and Analysis of Variance tests, with the scales serving as dependent variables and factors including condition, gender, and location used as independent variables. For all inferential statistics, normal distributions within the population were assumed allowing for the use of non‐parametric tests, while CI_95_ and correlation coefficients are reported in addition to p‐values.

The open‐text questions were analyzed using an inductive content analysis approach (Elo & Kyngäs, 2008; Erlingsson & Brysiewicz, 2017) to provide a description of the participant's experiences of the main changes to their contact with specialist healthcare providers, and the impact of the pandemic. This process involved familiarization with the data, developing meaning units from the responses, formulating codes, and generating categories and themes. The codes were developed by three researchers independently and iteratively refined collaboratively. The coded data and categories were discussed with the wider team and a consensus was reached on the categories and themes. Where indicative quotes are included participant identifiers are noted in parenthesis.

## RESULTS

A final sample of 147 individuals was included in the study (see Table 1). The sample was predominantly female (76.2%, *n* = 112), with male (21.1%, *n* = 31) and non‐binary (0.7%, *n* = 1). The age range of the sample was 19–84 years (*Mean* = 48.2, *SD* = 11.3), with 20% under the age of 40 years and 14.5% aged 60 and over. Just under two‐thirds of the sample were based in Ireland (62.2%, *n* = 92), with 33.3% based in the UK (*n* = 49) and 3.4% (*n* = 5) in other locations. Within these countries the participants were spread relatively equally across large urban (31.3%, *n* = 46), small urban (16.3%, *n* = 24), suburban (29.3%, *n* = 43), and rural (23.1%, *n* = 34) locations. In terms of the medical conditions reported by the participants, the majority of the group reported only one chronic condition (76.2%, *n* = 112), with 23.8% (*n* = 35) reporting two or three conditions. There was no evidence of systematic differences in demographic characteristics across the illness groups.

**TABLE 1:** Classification of illness frequency and age by group

Condition^a^	*n*	%	Mean age^b^	SD	Median age
Diabetes (Type 1 and Type 2)	43	29.3%	44.7	10.0	47.0
Rheumatology (IA and osteoarthritis)	36	24.5%	47.0	10.6	47.0
Cardiac (including heart failure)	20	13.6%	48.6	11.1	48.5
Respiratory (including asthma and chronic obstructive pulmonary disease)	12	8.2%	44.1	8.4	44.5
Multiple conditions	35	23.8%	54.8	11.8	52.0

a One participant reported an ‘other’ condition.

^b^ Those with multiple conditions were statistically significantly older than those with diabetes (*F* [4139] = −4.952, *p* = 0.003; *CI*_*95*_–17.680; −2.478).

Data on the length of the condition were available for 131 participants, with the timeframe ranging from under 1 to 71 years (*Mean* = 20 years, *SD* = 15.2). Overall, 27.2% of the sample (*n* = 40) had lived with at least one chronic condition for less than 10 years, while 38.1% (*n* = 56) had lived with at least one chronic condition for more than 20 years. Table 2 reports the length of illness by condition for any reports of the diagnosis (including those with multiple conditions).

**TABLE 2:** Frequency of condition and length of illness

Condition^a^	*N* (m)	Range^b^	Median	<1 year	1–5 years	6–10 years	>10 years
Diabetes	61 (3)	<1–54 years	24.5	4	11	2	41
Rheumatology	56 (26)	2–31 years	10.0	0	12	16	25
Cardiac	36 (6)	<1–71 years	3.0	8	12	3	7
Respiratory	34 (6)	2–61 years	25.0	0	2	2	24
Other	1 (1)	N/A	N/A	‐	‐	‐	‐

^a^ Includes all reports of a condition, so the total is >100%, with missing data noted (m).

^b^ Those with very extended periods of illness reflect congenital conditions, note some missing data.

### Engagement with healthcare

Participants were asked to report the number of healthcare contacts (both face‐to‐face and online) before and during COVID‐19 restrictions and reports were tabulated to identify any possible impact (see Tables 3 and 4). Cells marked in dark gray represent increases in contacts, while light gray reflects decreases. Cells appearing on the diagonal (no shading) reflect no change in reporting.

**TABLE 3:** Face‐to‐face contacts

	During COVID‐19 restrictions				
Before COVID‐19	One contact	2–5 contacts	6–10 contacts	>10 contacts	Total
One contact	17	5	1	1	24
2–5 contacts	30	26	1	3	60
6–10 contacts	1	5	0	0	6
>10 contacts	1	2	0	2	5
Total	49	38	2	6	95

**TABLE 4:** Non‐face‐to‐face contacts

	During COVID‐19 restrictions				
Before COVID‐19	One contact	2–5 contacts	6–10 contacts	>10 contacts	Tot
One contact	32	19	4	1	56
2–5 contacts	6	14	3	0	23
6–10 contacts	0	0	1	0	1
>10 contacts	0	1	0	0	1
Total	38	34	8	1	81

Of the 95 participants who answered both of these questions for face‐to‐face contacts (Table 3), just under half (47.4%, *n* = 45) reported no changes in contact, while 41.1% (*n* = 39) reported a drop in contacts and 11.6% (*n* = 11) an increase in contacts. Examining the same patterns for participants reporting non face‐to‐face contacts (*n* = 81, see Table 4), almost 60% (58.0%, *n* = 47) reported no changes in contact. However, 8.6% (*n* = 7) reported a drop in contacts while a third of participants (33.3%, *n* = 27) reported an increase in contacts.

Participants also self‐reported the perceived impact of COVID‐19 restrictions on their contact with a specialist healthcare provider. While 15% did not respond to this question, a further 15% (*n* = 22) reported that contact had increased, just over one‐third (35.4%, *n* = 52) reported no change, while a similar proportion (34.7%, *n* = 51) reported contact had decreased.

The qualitative data describe perceptions of changes in contact with healthcare providers among respondents, with reports of both increased and reduced frequency of consultations. Participants perceived that virtual care provided a better use of time, and reduced costs, however, there was an increased reliance on people to self‐report and a reduction in screening/surveillance. Participants also reported that availability and/or access to care was altered and more difficult, with a change in emphasis away from chronic illness. Some respondents indicated that not only were their healthcare providers less available to them, but there was also a lack of input into their care from the various members of the multidisciplinary team, a key feature of chronic illness management prior to the pandemic. This perception is illustrated in the following extract from one respondent:

“I feel we are being left behind, with covid being top priority as time goes on our conditions can get worse nobody knows unless regular checks are done. When covid was not here it was deemed necessary for checks regularly but now in covid it doesn't seem to matter”. (86)

For other respondents' remote access was considered positive, with indications that healthcare providers were actually more available and easier to access:

“My provider seems to be more available via telehealth than they were before the pandemic”. (119)

However, for others, the “remote environment' was less comfortable, and they felt their encounters were time limited”. (60).

Some unexpected consequences of virtual consultations were also reported with participations sharing that family members were able to participate in their consultation. For others virtual participation meant they had reduced traveling time and costs associated with attending in‐person appointments, which indicated that virtual contacts seemed more convenient:

“I had one telephone conversation with a nurse instead of a doctor and it was great ‐ I didn't have to pay for parking. I didn't have to wait for over an hour, they had looked at my notes previously and asked relevant questions. It was quite disappointing to go back into the clinic. I can understand if you have something that needs to be physically inspected, but I would love to have more of these kinds of consultations rather than face‐to‐face.” (184)

### Analysis of scale scores

Descriptive statistics for the scales used are reported in Table 5. For the Patient Assessment of Chronic Illness Care (PACIC), participants scored in the lower end of the possible range. In comparison, on the other scales, participants were showing a wider range of scores. There was no evidence of any statistically significant impact of demographic variables on scores on these scales.

**TABLE 5:** Descriptive and interferential statistics for standardized scales.

Scale	Mean	SD	Actual range	Possible range	Published mean (SD)
Self‐care of chronic illness inventory^a^					
A: Common self‐help behaviors	68.8	11.8	37.5–93.7	0–100	74.3
B: Common monitoring behaviors	72.2	19.4	20–100	0–100	77.9
B: Recent monitoring behaviors	75.3	20.4	25–100	0–100	N/A
C: Common control behaviors	62.2	17.3	20–100	0–100	67.6^b^
Self‐care self‐efficacy scale	37.0	8.65	10–50	10–50	N/A
Patient assessment of chronic illness care total^c^	2.37	0.9	1–4.6	1–5	3.2 (0.9)
PACIC—Patient activation^c^	2.81	1.2	1–5	1–5	3.6 (1.1)
PACIC—Decision support^c^	2.69	0.9	1–5	1–5	3.5 (0.9)
PACIC—Goalsetting & Tailoring^c^	2.17	0.9	1–5	1–5	3.0 (0.9)
PACIC—Problem solving^c^	2.62	1.1	1–5	1–5	3.4 (1.1)
PACIC—Follow‐up/Coordination^c^	1.94	0.9	1–4.6	1–5	2.9 (1.0)
Coronavirus impact scale	17.7	3.77	8–27	8–32	N/A

^a^ Scale Scores have been standardized scale (0 = 100) and compared to figures reported by van Rijn et al (in press) for a sample with heart failure.

^b^ van Rijn et al. reported control behavior scores for people with and without symptoms, this figure represents the average across the two groups.

^c^ Calculated as an average score—Published means from Glasgow, Whitesides, et al., 2005 reporting for the sample with diabetes.

For the *PACIC scale and subscales*, the present sample scored statistically significantly lower than the sample of people with diabetes reported by Glasgow, Whitesides, et al. (2005) ‐ PACIC Total t (97) = −9.074, *p* < 0.001, CI95–1.0104, −0.6477; PACIC Activation t (101) = −6.763, *p* < 0.001, CI95–1.0169, −0.5557; PACIC Decision Support t (99) = −8.157, *p* < 0.001, CI95–1.0029, −0.6104; PACIC Goalsetting t (99) = −8.546, *p* < 0.001, CI95–1.0174, −0.6340; PACIC Problem‐solving t (101) = −7.147, *p* < 0.001, CI95–1.0026, −0.5670; and PACIC Follow‐up t (100) = −10.587, *p* < 0.001, CI95–1.1298, −0.7732. A similar pattern was observed for the *Self‐Care of Chronic Illness (SCCI) subscales*, with the present sample scoring statistically significantly lower than a sample of people with heart failure (van Rijn et al., 2022)—SCCI Common Self‐Help t (112) = −4.948, *p* < 0.001, CI95‐7.6948, −3.2945; SCCI Common Monitoring t (112) = −3.135, *p* = 0.002, CI95‐9.3539, −2.1098; SCCI Recent Monitoring N/A; Common Control t (112) = −3.346, *p* = 0.001, CI95‐8.6479, −2.2158. It is also noted that the sample in the present study scored below the cut‐off of 70 reported by van Rijn et al. (2022) for both self‐care and control subscales.

The perceived impact of COVID‐19 generally showed weak correlations with self‐care management and monitoring, however, there was a small negative correlation with the self‐care self‐efficacy scale (*r* = −0.352; *p* < 0.001). In addition, small negative correlations were found with all subscales of the PACIC (*r* values from −0.229 to −0.308; *p* < 0.015 for all correlations), with the exception of the activation subscale (*r* = −0.166; *p* = 0.055). The direction of the albeit weak correlations suggests that the higher impact of COVID‐19 was associated with lower self‐efficacy/confidence and lower assessments of care.

Respondents echoed these findings in the open‐text questions on the impact of the pandemic on life, indicating that public health recommendations for isolation and restriction of movements had negative consequences. Two themes illustrate this, the “emotional response” and “feeling restricted.” Emotionally, respondents expressed impacted their reduced sense of confidence; “Hope I get my confidence back (87).” This reduced sense of confidence also impacted their engagement in clinic consultations with a respondent reporting they did not feel they could rely on their own judgment or distinguish what was relevant in relation to their own healthcare:

“I have been critically ill which has necessitated a lot more interaction. I have had all care with my usual provider over the telephone which has been more easy to discuss properly and in detail than in the past face‐to‐face but does rely more on my judgement. This is stressful as I have no real measures or knowledge to know whether I'm making the right judgement calls and they are trusting and relying on me to do that.” (43)

The emotional responses also include a sense of fear, grief, and isolation with a particular fear of healthcare settings and of catching COVID‐19.

In relation to feeling restricted respondents reported feeling isolated related to loneliness, lack of social contact, and the need to attend alone for care. They also illustrated challenges when considering re‐engagement with society having led a more insulated life during the pandemic:

“Has made me even more insular ‐ I must be the only one who is quite happy with quarantine, although my confidence, work, socialisation skills etc have suffered, I'm not normal am I? “(45)

Feeling restricted, has also had financial impacts and some increased workload due to responsibilities with homeschooling. While for others feeling restricted was more positive, in relation to self‐management and virtual consultations.

## DISCUSSION

The results of this research identified that the reported experiences of access to healthcare providers were similar for people with chronic illnesses irrespective of their primary diagnosis. This research utilized a combination of quantitative and qualitative data, which was uncommon in COVID‐19 research. There are two key findings evident in this study. The first is that while there were negative correlations between the impact of COVID‐19 and both confidence in self‐care and assessment of care these were low. The second is that the majority reported no change or an increase in face‐to‐face contact with their healthcare providers. While the amount of contact may not have changed their assessment of the quality of care reflected negative experiences. The quantitative data are supported qualitatively in responses relating to the two key findings.

This research suggests that healthcare support during COVID‐19 restrictions was associated with reduced self‐care behaviors for people with chronic illnesses. Participants in this research reported reduced support from the chronic illness team which was associated with a negative impact on their ability to perform self‐care. Chronic illness healthcare services are delivered primarily by specialist nurses. Mobilization of nurses throughout the pandemic relied heavily on the redeployment of specialist nurses (Ryder et al., 2022). It is reasonable, therefore, to expect that their redeployment would result in a negative impact on chronic illness services. Forde et al. (2021) reported that specialist nurses identified distress for people with diabetes across Europe with reduced support resulting in increased complications. Reduced support for people described here specifically impacts self‐care education and support.

This research suggests that higher impacts of COVID‐19 were associated with lower confidence and lower assessment of care, though the magnitude of the association suggests that other factors may have been more influential. Participants reported a lack of confidence in self‐monitoring and symptom interpretation when contributing to virtual consultations. Leese et al. (2021) have reported that some people with rheumatoid arthritis experienced an improvement in their self‐care practices while remaining at home. However, Massouh et al. (2020) reported that self‐efficacy/confidence is a key determinant in self‐care, enhanced by social support and knowledge.

Assessment of care is taught by specialist nurses when teaching self‐care. Teach‐back is a communication method recommended to confirm that people understand the information provided about their condition (Hong et al., 2020). The preferred mode for education by healthcare providers is face‐to‐face, with limited training to educate people via telephone. There is a dearth of literature reporting on virtual education for patients, however, one randomized controlled trial for Asthma (Nguyen et al., 2008) deduced that self‐management was comparable when educated via the Internet versus face‐to‐face. Nguyen et al. (2008) provided a structured developed internet program for participants, which is not comparable to ad hoc telephone education delivered by a reduced workforce during COVID‐19 restrictions. Healthcare staff had limited orientation to deliver equivalent education for people with chronic illness using alternative virtual mediums during the pandemic. The previously mentioned COVID‐19 restrictions removed social support for many people and simultaneously reduced quality of access to healthcare services and more importantly specialist nurses to provide self‐care knowledge for people with chronic illnesses.

This research also provides a unique insight into the perceived effect of self‐care for people with a chronic illness during COVID‐19 restrictions. Previous research has reported increased stress levels for people with chronic illnesses (Pawłowski et al., 2021) and increased anxiety levels for older people with a chronic illnesses (Ozteke Kozan & Kesici, 2021). In addition, Ciacci and Siniscalchi (2020) reported people had discontinued medications during the pandemic, but their primary concern was fear. The research highlighted widespread delayed access to healthcare worldwide affecting people with chronic illnesses (Aktas, 2021; Gualano et al., 2021; Sankaranarayanan et al., 2021; Zukowski et al., 2021). Consequently, healthcare professionals were urged not to forget people with chronic illnesses (Mauro et al., 2020) and to utilize telemedicine as an alternative to face‐to‐face healthcare. This research suggests there were mixed perceptions related to contact with healthcare providers, leaving people with chronic illnesses feeling they were forgotten. The findings echo previous research (Aktas, 2021; Pawłowski et al., 2021; Sankaranarayanan et al., 2021; Zukowski et al., 2021) related to individual illness and build on this to conclude experiences for people were similar irrespective of the disease.

The research supports healthcare provider perceptions that people with chronic illness experienced a reduced quality of access to healthcare support during the COVID‐19 pandemic. The findings also highlight that careful consideration and preparation are required in advance of transferring chronic illness healthcare provision to remote services.

### Lessons learnt due to Covid‐19 pandemic and policy change proposals

The lessons learnt from this research related to the COVID‐19 pandemic are that people with chronic illness, irrespective of their diagnosis, expressed a reduction in access to quality healthcare. In the event that future healthcare policy promotes virtual healthcare, people with chronic illnesses are appropriately prepared for remote monitoring. Future patient education should include focused self‐assessment and symptom interpretation. In addition, chronic illness teams must maintain some levels of support for people attempting to manage at home despite escalating hospital pressures. Future policy should include plans and procedures to maintain chronic illness support services during periods of escalation.

There are some strengths and limitations evident in the research that should be considered before a conclusion is drawn. The use of an online survey ensured accessibility to a wider population of potential participants, and this was supported by the use of social media recruitment. However, the achieved sample is significantly smaller than was hoped and there are clear implications for the representativeness of the findings. Social media recruitment arguably targeted a younger sample, therefore transferability of the findings to older people with a greater prevalence of chronic illness must be considered. Despite this concern, the findings do provide some insight into the experiences of people with chronic illness during a period of COVID‐19 restrictions, within the parameters of these limitations.

## CONCLUSION

This research highlights the importance of providing continued support to the higher risk population, including people with chronic illness irrespective of other challenges to healthcare services. In addition, a purposeful approach to preparing people with chronic illness is recommended to ensure they are adequately prepared to perform self‐care supported with remote monitoring rather than face‐to‐face healthcare.

### Clinical resources

- Self‐Care of Chronic Illness inventory.

https://self‐care‐measures.com/available‐self‐care‐measures/self‐care‐of‐chronic‐illness‐inventory/

## CONFLICTS OF INTEREST

The authors wish to declare that there are no conflicts of interest.
